# Broad complex tachycardia; never judge a book by its cover

**DOI:** 10.1007/s12471-020-01495-x

**Published:** 2020-10-02

**Authors:** M. V. Regeer, L. F. Tops, M. de Riva Silva

**Affiliations:** Department of Cardiology, Leiden Heart-Lung Center, Leiden University Medical Center, Leiden, The Netherlands

A 69-year-old male presented to the emergency department with broad complex tachycardia (Fig. 1). He had been diagnosed with non-ischaemic cardiomyopathy with complete left bundle branch block (LBBB) and a left ventricular ejection fraction (LVEF) of 20% ten years earlier, for which he received cardiac resynchronisation therapy with defibrillator function (CRT-D). He was a good responder with LVEF improving to 49% and functioning in New York Heart Association (NYHA) functional class I. A recent outpatient clinic electrocardiogram (ECG) showed sinus rhythm with biventricular pacing (Fig. 2).

**Fig. 1:**
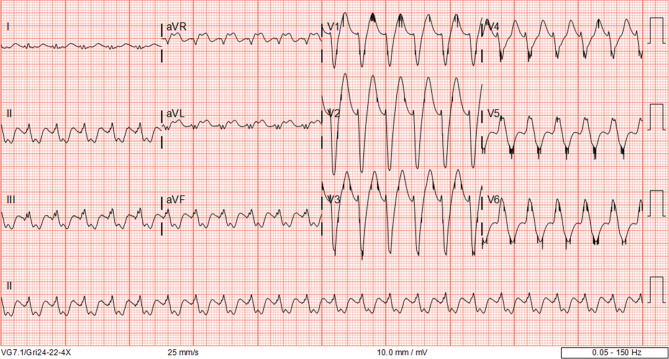
Electrocardiogram of broad complex tachycardia

**Fig. 2:**
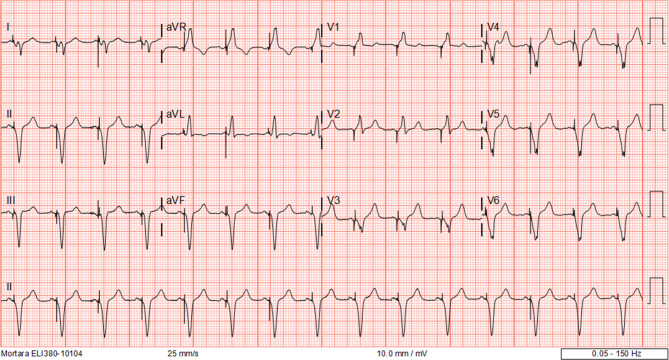
Outpatient clinic electrocardiogram with biventricular pacing

On the day of admission he woke up with palpitations after an afternoon nap. He denied chest pain, dyspnoea and dizziness. Upon arrival of emergency medical services he was haemodynamically stable with a blood pressure of 124/76 mm Hg and a broad complex tachycardia of initially 200 bpm, slowing down to 138 bpm after 150 mg amiodarone.

What is the origin of the broad complex tachycardia?

## Answer

You will find the answer elsewhere in this issue.

